# Fabrication and Optical Properties of Strain-free Self-assembled Mesoscopic GaAs Structures

**DOI:** 10.1186/s11671-016-1782-1

**Published:** 2017-01-21

**Authors:** Saimon Filipe Covre da Silva, Thayná Mardegan, Sidnei Ramis de Araújo, Carlos Alberto Ospina Ramirez, Suwit Kiravittaya, Odilon D. D. Couto, Fernando Iikawa, Christoph Deneke

**Affiliations:** 1 Laboratório Nacional de Nanotecnologia (LNNano/CNPEM), 13083-100 Campinas, SP Brazil; 2 Departamento de Física, Universidade Federal de Viçosa, 36570-900 Viçosa, MG Brazil; 3 Universidade Federal de Itajubá, Campus Itabira, 35903-087 Itabira, MG Brazil; 4 Department of Electrical and Computer Engineering, Faculty of Engineering, Naresuan University, Phitsanulok, 65000 Thailand; 5 Instituto de Física “Gleb Wataghin”, Universidade Estadual de Campinas, 13083-859 Campinas, SP Brazil

**Keywords:** Strain-free quantum dots, Local hole etching, Ga-assisted deoxidation, Molecular beam epitaxy

## Abstract

We use a combined process of Ga-assisted deoxidation and local droplet etching to fabricate unstrained mesoscopic GaAs/AlGaAs structures exhibiting a high shape anisotropy with a length up to 1.2 μm and a width of 150 nm. We demonstrate good controllability over size and morphology of the mesoscopic structures by tuning the growth parameters. Our growth method yields structures, which are coupled to a surrounding quantum well and present unique optical emission features. Microscopic and optical analysis of single structures allows us to demonstrate that single structure emission originates from two different confinement regions, which are spectrally separated and show sharp excitonic lines. Photoluminescence is detected up to room temperature making the structures the ideal candidates for strain-free light emitting/detecting devices.

## Background

Semiconductor quantum dots have been the subject to intensive research in the last three decades and one of the model systems for nanotechnology. One of the main mechanisms to fabricate these nanometer-sized structures is the Stranski–Krastanov growth mode of epitaxial lattice mismatch materials [1–3]. This method has the disadvantage that the obtained quantum dots are inherently strained, which modifies optical and electrical properties of the material [4]. In the last decade, several strategies have been developed to overcome this restriction and to obtain strain-free quantum dot structures [5–15]. One common strategy is the use of thickness fluctuations of an AlGaAs/GaAs/AlGaAs quantum well (QW) [13–15]. A second tactic is to pattern the substrates with holes and then grow a light-emitting structure of AlGaAs/GaAs/AlGaAs on top resulting in a quantum dot at the hole position in the initial template. Strategies for hole fabrication have included the in situ etching of holes [16, 17] and their overgrowth [7], Ga-assisted hole etching and filling [9, 11, 12, 18–23], or overgrowth of patterned substrates [8]. These strain-free structures constitute an own class of nano-sized optical emitters and have been used successfully in a variety of fascinating experiments and applied device structures such as studying of the exciton–exciton and exciton–phonon interaction in quantum dot ensembles [24], coherent optical controlled quantum states [25], single-photon sources [26–28], entangled photon sources [29], and tuning the optical emission by local straining [30, 31] or the optical control of the nuclear spin for potential quantum-computing applications [32–34].

Common current approaches share that the produced GaAs dots are rather shallow and it is hard to realize a good confinement. For dots defined by thickness fluctuations, this problem is intrinsic to the fabrication and the use of two-dimensional islands of the epitaxial growth [13]. When defining the hole template by etching, these holes tend to be shallow preventing the growth of thick lower AlGaAs barriers [7]. Alternatively, the bottom barrier is grown first and then structured by in situ etching, introducing a growth interruption exposing an AlGaAs surface [9, 12, 23]. Extended thick GaAs structures are commonly not found, even though they would offer an additional tuning parameter, broadening the optical and physical properties.

In this work, we fabricate a room temperature active elongated mesoscopic GaAs structure (MGS) with a high shape aspect ratio. The MGS have been made by molecular beam epitaxy combining Ga-assisted deoxidation [35, 36] with local hole etching [10, 18, 19, 22] in a machine equipped with a thermal As source. Our morphological study by atomic force microscopy (AFM) finds an average hole depth of 19 nm and ca. 200 nm hole diameter. The holes exhibit crystal facets as side walls making them hexagonally shaped. They are surrounded by GaAs mounds. By overgrowing these holes with different amounts of Al_0.33_Ga_0.67_As, the shape and size of the initial template can be tuned. We demonstrate that layers as thick as 20 nm can be grown without significantly decreasing the hole depth and sizes, reproducing the elongated mound surrounding the hole. We fill this hole-mound structure with different amounts of GaAs and then cap it with another upper 20-nm Al_0.33_Ga_0.67_As barrier. Studies by transmission electron microscopy (TEM) of focused ion beam (FIB) prepared cross-sections together with AFM results allow us to develop a complete structural image of the MGS. Microphotoluminescence (μ-PL) investigations reveal room temperature (RT) optical emissions of the MGS. At low temperature, the PL spectrum is composed of closely spaced single excitonic lines in an energy range of 1.58 to 1.72 eV and the contribution of the 15-nm-deep hole-mound structures at ca. 1.55 eV. Temperature-dependent μ-PL investigation indicates an electronic coupling of these energetically closely spaced lines creating a unique mesoscopic system compromising a single dot formed by the deep hole coupled to thickness fluctuation dots formed by the elongated mound structure. Hence, the mesoscopic GaAs structures form a quantum dot system with high potential for application in optical devices.

## Methods

All the samples were grown in a 2-inch custom-made solid-state molecular beam epitaxy machine (Dr. Eberl MBE-Komponenten GmbH) at LNNano (CNPEM, Brazil). Growth is monitored in situ by reflective high-energy electron diffraction (RHEED). The chamber uses a thermal As_4_ cell opened and closed by a linear shutter that reduces the beam equivalent pressure (BEP) by ca. 100 times (1.2 × 10^−5^ to 2.7 × 10^−7^ mbar). This kind of cell needs, compared to valve cracker cells, some time to reach and stabilize the As_4_ flux for a given temperature—for our machine ca. 15 min for typical temperature of 314 °C. During this stabilization time, the As flux continually increases non-linearly and finally saturates at its maximum value.

GaAs (001) wafer pieces with a previously grown GaAs buffer were In-glued on the 2″ Si wafer and introduced into the MBE and preheated at 300 °C. Hole templates were fabricated by a combination of Ga-assisted deoxidation and local Ga hole etching [10, 18, 22, 35, 36]. In the absence of an As counter pressure, the sample was exposed repeatedly 20 times to a Ga flux of one monolayer (ML) per minute for 30 s followed by a 30-s growth interruption at a substrate temperature of 450 °C. A streaky RHEED showed up after six to seven ML indicating a complete removal of the native oxide layer. During the Ga-assisted deoxidation process, we ramped the As cell to 290 °C corresponding to an increase of the low background pressure from 2 × 10^−9^ mbar to 1 × 10^−8^ mbar. After finishing the initial Ga deposition, the As cell temperature is raised to 314 °C and the gallium rate is increased to ~0.3 μm/h. The sample is annealed for 5 min during the time that the As cell needs to reach 314 °C with the shutter closed.

With the As cell shutter closed, we deposited Ga of an 5 nm equivalent GaAs thickness during 1 min and opened afterwards the As shutter. A further annealing of 15 min was carried out to stabilize the As counter pressure—the chamber background pressure rised from 2 × 10^−8^ mbar to 1.5 × 10^−6^ mbar (corresponding to a BEP of 2 × 10^−5^ mbar stabilizing a rate of ca. 0.4 ML/s for our machine). This process resulted in the formation of a hole surrounded by a long mound structure on an otherwise flat GaAs (001) surface.

On a typical hole-mound structure of the GaAs surface described above, we deposited Al_0.33_Ga_0.67_As layers with different thickness to study the hole filling at the same substrate temperature (450 °C) used for the hole creation. In the second set of samples, we chose a particular template containing hole-mound structures with a 20-nm AlGaAs layer and deposited different GaAs layer thicknesses by pulsed epitaxy [12, 22] with a growth rate of 0.3 μm/h. The GaAs was deposited at 450 °C substrate temperature opening the shutter for 12 s (1 nm) and waiting for 36 s. The number of repetitions was tuned according to the nominal GaAs thickness for filling. Samples were annealed for 10 min ramping the substrate to 570 °C after GaAs deposition. In the third set ones, for photoluminescence studies, a top barrier of 20-nm Al_0.33_Ga_0.67_As layer followed by a 5-nm GaAs cap were grown at 570 °C.

After every growth step, reference samples were taken out of the chamber and the surface studied by AFM. We used a NX10 instrument (Park System) in tapping mode with standard AFM cantilevers. Images were post-processed and statistical analysis carried out with the SPM software Gwyddion. For the holes, structures were measured using the line scan tool of the software, whereas the mesoscopic GaAs structures were analyzed using the grain finding function with the statistical analysis module.

A cross-section preparation was carried out with a FEI Helios NanoLab 660 instrument of the LNNano (CNPEM, Campinas). First, a 300-nm electron beam-assisted deposition carbon layer followed by a 300-nm electron beam-assisted deposition platinum layer and a 3-μm Gallium beam-assisted deposition Pt layer were placed to protect the mesoscopic GaAs structure during the preparation. Two 15 × 12.4 μm and a 15 × 7 μm slices were cut out of the GaAs sample using a high-energy Ga beam, perpendicular to the [110] and [1–10] zone axes, respectively. Using a nanomanipulator, the slices were then transferred and attached to a TEM grid. After this, the slices were further thinned down to ca. 50 nm to ensure transparency in the TEM investigations.

TEM analyses were conducted in a JEOL JEM 2100F microscope (LNNano/CNPEM, Campinas). The instrument was operated under 200 kV with a field emission gun running at a current of 146 μA in scanning transmission electron microscopy mode (STEM). To achieve a good chemical contrast, a high-angle annular dark-field (HAADF) detector was used.

The μ-PL measurements were carried out at a homebuilt setup using 20 and 100× objective lenses which produce laser spot sizes of approximately 80 and 2–3 μm^2^, respectively. The setup uses a green solid-state diode laser with a wavelength of 532 nm. Samples were mounted in a cold-finger He-cryostat and spectra were acquired by a ½-m single spectrometer coupled with a Si-CCD camera (Horiba).

## Results and Discussion

**Fig. 1:**
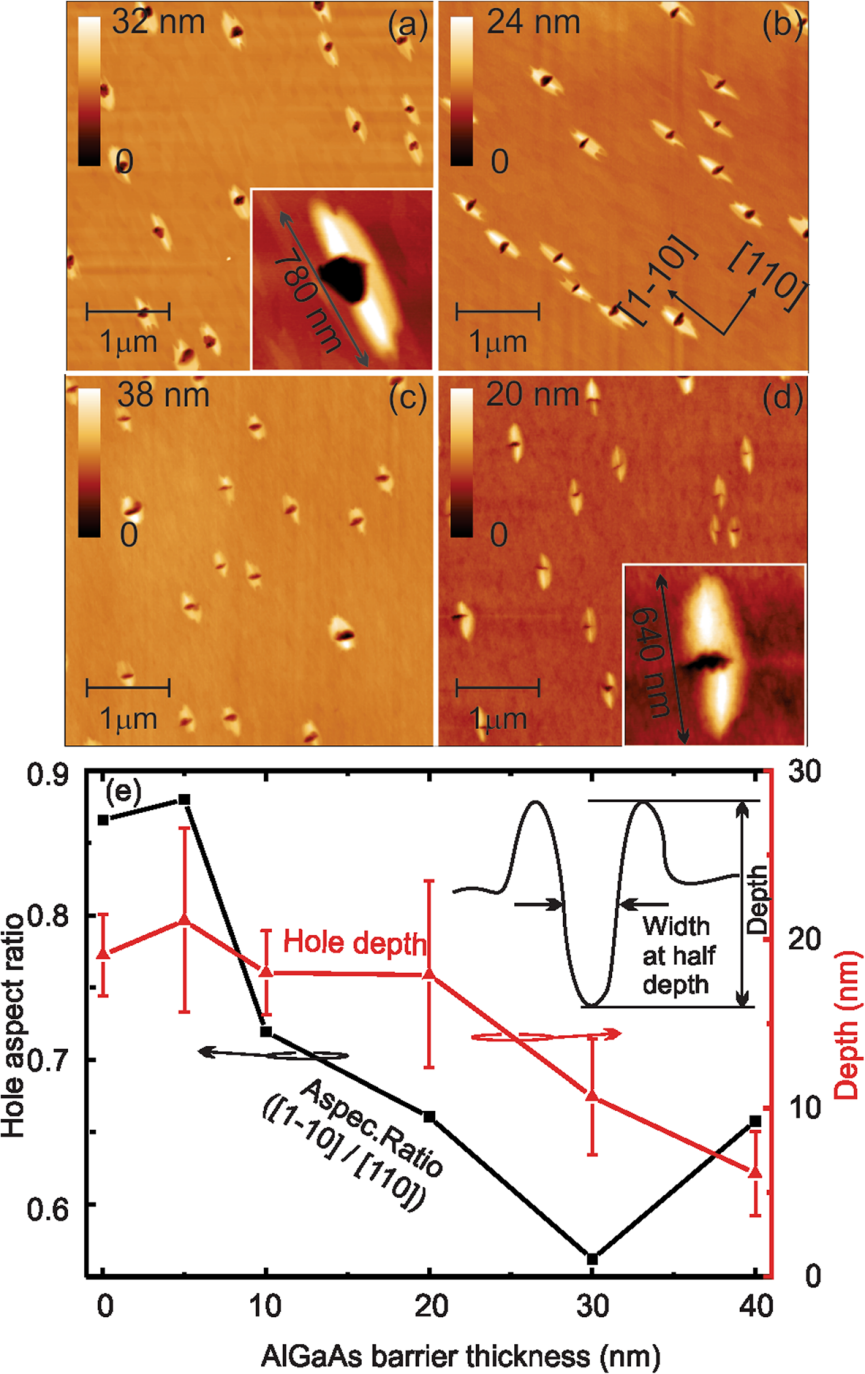
AFM images showing the initial holes and the hole evolution with different Al_0.33_Ga_0.67_As deposition thicknesses. **a** AFM image of the initial holes as fabricated. A GaAs mound elongated into the [1–10] direction surrounds the holes. The *inset* shows a single hole-mound with a length of ca. 780 nm and a width of ca. 200 nm. **b**–**d** AFM images of the initial hole-mounds with 5, 20, and 40 nm Al_0.33_Ga_0.67_As deposited. The *inset* in (**d**) shows that the hole-mound with elongated in the direction [1–10] is preserved after the Al_0.33_Ga_0.67_As deposition. **e** Morphological study of the hole depth and the hole aspect ratio measured at the half hole depth as a function of the AlGaAs thickness. The *inset* shows the schematic hole-mound profile

Figure 1 shows overview AFM images of 5 × 5 μm^2^ corresponding to the creation of the initial hole template and the filling with AlGaAs.

Figure 1a shows a surface morphological AFM image of the holes fabricated. The normal GaAs surface appears flat exhibiting GaAs single monolayer steps. We can identify holes surrounded by elongated mounds, which we named hole-mound structure in the following. From the AFM image, we deduce an average hole-mound density of 1.0 ± 0.2 μm^−2^. This relatively low density is required to study a single structure by μ-PL technique. A typical single hole-mound structure is shown in the AFM image of the Fig. 1a inset. The mound is elongated into the [1–10] direction with a length of ca. 780 nm and a width of ca. 200 nm. The mound formation and evolution arises from the anisotropic Ga adatom diffusion on top of GaAs surface [7, 20, 37, 38]. The main feature of the mound structures is their large extension along the [1–10] direction, which we attribute to our stepwise Ga deposition and long annealing process under an increasing As flux. The long mound is only observed under long annealing time and, surprisingly, the hole size is not affected significantly, which is an advantage for optical applications. The hole in the middle of the mound has a depth of 19 nm representing the average of the holes seen in the 5 × 5 μm^2^ overview AFM image of Fig. 1a. In the detailed image shown in the inset, the hole appears not round but exhibiting facets. We ascribe faceting of holes defined in a GaAs surface to the formation of low energy surfaces during the annealing of the hole in an As flux [39]. The holes are slightly asymmetric at the top with a diameter of 190 and 230 nm in the two crystal directions.

Figure 1b–d illustrate the evolution of the hole filling with 5, 20, and 40 nm of Al_0.33_Ga_0.67_As by AFM images of the surface obtained right after material deposition. The AFM image shown in Fig. 1b depicts the surface morphology of the initial hole template covered with 5 nm Al_0.33_Ga_0.67_As. In the image, the hole-mound structure surrounded by a flat layer can be identified exhibiting only monolayer high steps. Comparing the hole-mound structures to the initial one in Fig. 1a, no significant changes are visible. The holes roughly keep their shape and the average hole depth stays unchanged. Furthermore, no changes of the mound structure are observed. This indicates that the initial 5-nm Al_0.33_Ga_0.67_As layer covers the surface and copies the underlying morphology.

Figure 1c shows an AFM image of a sample with 20-nm Al_0.33_Ga_0.67_As layer. The holes have an average depth of 17 ± 5 nm and start to close, losing the clearly defined facets observed in Fig. 1a (also illustrated in the drawing in Fig. 3a). Interestingly, the closing starts along the [1–10] direction, whereas the hole width does not change in the [110] direction. The mound undergoes morphological alterations and appears slightly more extended into the [110] direction.

To finalize the morphology evolution study, a sample with 40-nm Al_0.33_Ga_0.67_As deposition is investigated (Fig. 1d). The holes are about to be fully filled. It is obvious from the image that material deposition almost closes the hole from the [1–10] direction, whereas the perpendicular [110] direction stays unchanged. The holes have an average depth of 6 ± 2 nm. The surrounding mounds seem in their extension comparable to the mounds seen in Fig. 1c. The asymmetric closing of the hole is illustrated with the AFM image of a single hole-mound structure depicted in the inset of Fig. 1d. Whereas the extension of the hole in the [110] direction is with 220 nm comparable to the extension of the initial hole (see inset in Fig. 1a), the hole has shrunken in the [1–10] direction and is after the 40-nm Al_0.33_Ga_0.67_As deposition only ca. 100 nm wide.

To study the changes of the holes during material deposition more quantitatively, the hole depth and hole aspect ratio are plotted in Fig. 1e as a function of the AlGaAs deposition. The black square mark corresponds to the aspect ratio of the hole width at half hole height (see inset Fig. 1e) in the two main directions (width in [1–10] divided by the width in [110]), and red triangles are for the depth of the holes (as marked in the inset of Fig. 1e). Lines are guides to the eye.

The hole depth (black squares) stays at quasi unchanged for the first 20 nm of AlGaAs deposition. Only for larger AlGaAs layer thickness, the depth reduces continuously and the hole gets shallower. This supports our statement that thin AlGaAs layers copy the underlying morphological structure—an effect observed before and ascribed to the low diffusion coefficient of AlGaAs on a GaAs surface [40]. As pointed out during the discussion of the AFM images in Fig. 1a–d, the hole morphology (as well as the mound morphology) undergoes an asymmetric filling. This is reflected in the shape aspect ratio (black squares) plotted in Fig. 1e. The hole aspect ratio is nearly 1 for the unfilled holes and stays close to 1 for a hole filling up to 5 nm. Then it continuously drops to lower values indicating that the hole gets smaller in the [1–10] direction due to the mound growth in this direction. We attribute this behavior to a preferred material diffusion and accumulation in this crystal direction.

From this morphological study, we decided that a 20-nm Al_0.33_Ga_0.67_As filling of the hole is an ideal template for light-emitting strain-free GaAs structures. For this thickness, the hole is still in average 17 nm deep—deeper than comparable templates for overgrowth described in literature. The aspect ratio is still close to one (it dropped from 0.9 to 0.7 in average) indicating that the holes are approximately round and do not exhibit a large asymmetry. Finally, 20 nm of Al_0.33_Ga_0.67_As is sufficiently thick to prevent carriers in any quantum structure made out of AlGaAs/GaAs from leaking out.

**Fig. 2:**
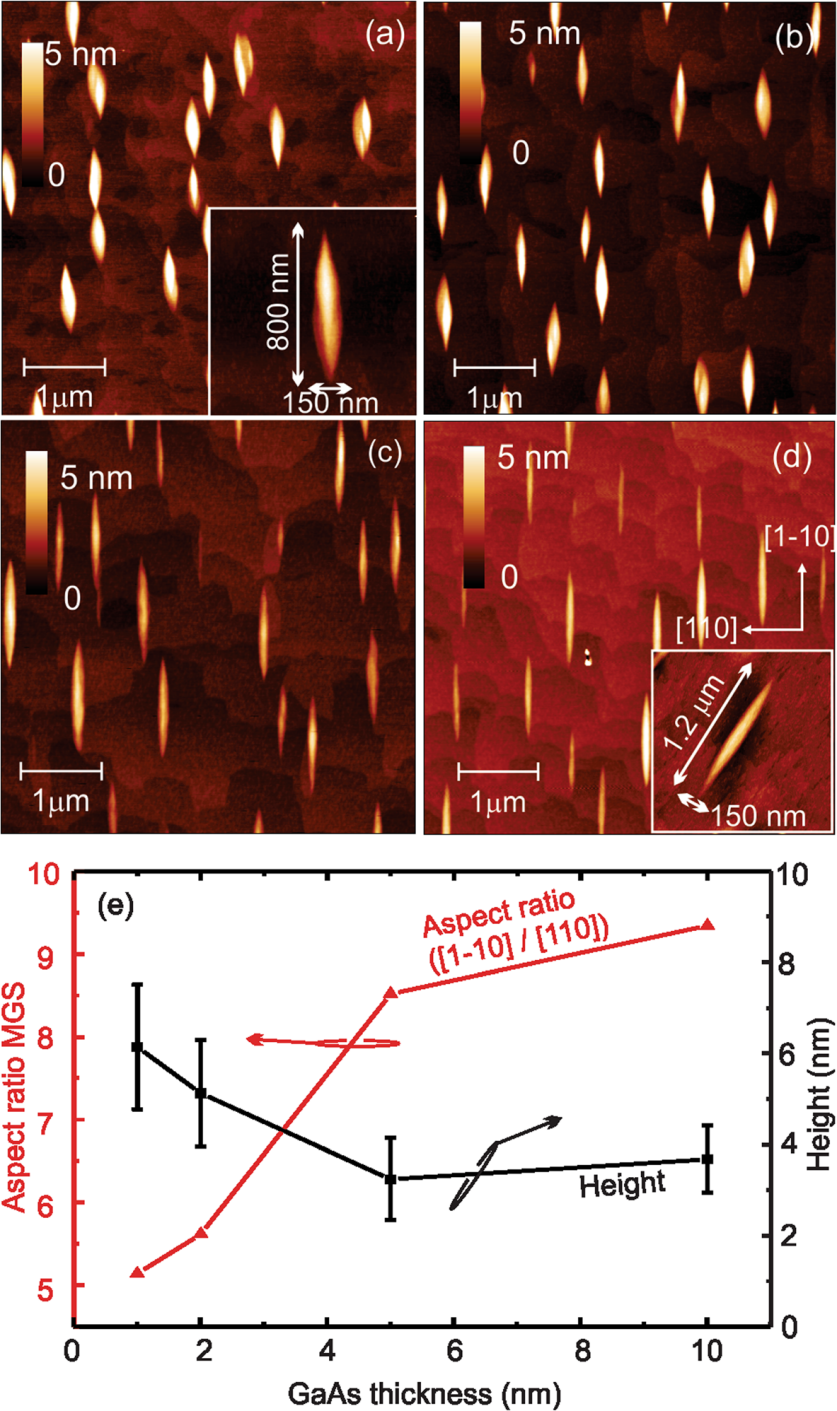
AFM 5 × 5 μm^2^ overview images of the GaAs deposition onto the 20-nm Al_0.33_Ga_0.67_As-filled hole used as a template. **a**–**d** AFM images of 1, 2, 5, and 10 nm of GaAs covered samples. We obtain elongated MGS aligned with the [1–10] direction. The *insets* in (**a**) and (**d**) show a magnification of the mesoscopic structure. **e** Morphological study (aspect ratio, area, and height) of the MGS evolution with increasing GaAs deposition. *Lines* are guides for the eye

For fabrication of a strain-free GaAs emitter, we follow the approach as described in detail in the experimental section defining a lower barrier (20 nm Al_0.33_Ga_0.67_As in our case), filling the hole-mound structure with GaAs, and finally capping the structure with 20 nm Al_0.33_Ga_0.67_As and a 5-nm GaAs top layer. Hereby, the amount of deposited GaAs for hole-mound filling will determine the characteristics of the emitter. To understand the hole-mound filling and study the obtained GaAs structures, a series of samples with different amounts of GaAs were grown. The development of the hole-mound filling is documented by the AFM images in Fig. 2 (without the upper 20-nm Al_0.33_Ga_0.67_As/5-nm GaAs cap).

Figure 2a shows the surface morphology by AFM after a 1-nm deposition of GaAs on top and post-annealing of a 20-nm Al_0.33_Ga_0.67_As template similar to that depicted in Fig. 1c. We observe the formation of larger mound structures on the assumed position of the hole-mound template and the hole is no longer observed, i.e., the hole is completely filled with GaAs due to the large Ga diffusion in this growth condition. The average height of the mounds is determined to 6 ± 2 nm by statistical analysis over the ensemble. As illustrated in the inset of Fig. 2a, representing an AFM image of a typical single mound structure, the GaAs mounds have a length of 800 nm in the [1–10] direction and a width of 150 nm in the [110] direction. Hence, they are much larger than the commonly found GaAs quantum dots made by in situ AsBr_3_ etching [7] or local hole etching [12, 22, 23] and do not constitute a nanostructure anymore. Therefore, we prefer to call them a mesoscopic GaAs structure (MGS), which we expect to have different physical properties compared to normal small GaAs dots previously described in literature. We ascribe the formation of these strain-free MGS to the influence of the underlying hole-mound template, which is already elongated in the same crystal direction. During GaAs deposition and annealing, the material preferentially attaches to these elongated sides of the mound amplifying growth into the [1–10] direction—the direction of the fast Ga diffusion on the GaAs surface [1].

With increasing amounts of GaAs deposition, (2, 5, and 10 nm), the MGS appears more elongated with a length of ca. 1 μm in the [1–10] direction, whereas the width along [110] is unchanged and their height slightly decreases to ca. 4 nm in average (see AFM in Fig. 2b–d). The longest MGS is shown in the inset of Fig. 2d where the length is 1.2 μm in the [1–10] direction and 150 nm in the [110] one. Hence, the structure increased by a factor of 1.5 in the [1–10] direction, keeping the same size in the [110] direction.

The statistical analysis of different parameters as a function of GaAs deposition is summarized in Fig. 2e. The black squares represent the average height of the mesoscopic structure, and the aspect ratio is presented by red triangles. With increasing GaAs layer thickness covering the hole-mound structures, the average height decreases from 6 to 3.6 nm, following the tendency to smooth the surface. The MGS aspect ratio of the longest to the shortest size of the structure increases from 5 to 9 illustrating that the [1–10] direction grows faster than the [110] direction. Furthermore, we observe a MGS evolution from a quasi-elliptical shape into a more elongated one. This confirms our assumption that the mesoscopic structure originates from the asymmetric Ga surface diffusion resulting in an increased growth rate in the [1–10] direction of the crystal. Hence, the MGS does not symmetrically flatten out as naively expected, but flattens by elongation in one direction and by continuously decreasing the mound height over the surrounding flat GaAs surface.

It is worth pointing out that we can use the morphological study of the holes depicted in Fig. 1e to estimate the material needed to fill the hole and obtain the structures observed and investigated in Fig. 2. Assuming a cylindrical hole with a radius of 100 nm and a depth in the range of 15 nm (overestimating the real hole-mound volume), ca. 0.5 nm or 1.5 ML of GaAs deposited in one square micrometer is needed to fill the hole for our hole density of ca. 1 μm^−2^.

**Fig. 3:**
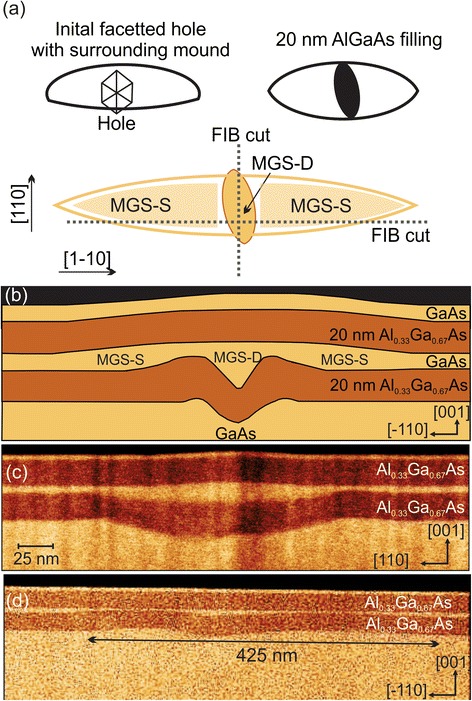
**a** Two top view schematic outlines of the initial hole-mound structure of GaAs surface (*left diagram*) and after covering with a 20-nm Al_0.33_Ga_0.67_As layer (*right*). The third diagram is the top view of the MGS covered with a 20-nm top Al_0.33_Ga_0.67_As layer and 5-nm GaAs, indicating two possible regions where the optical emissions occur. The *dotted lines* show the position where the structure was cut for HAADF-STEM analysis. **b** Illustration of the cross-section view of the structure. **c** HAADF-STEM image (false color scale to enhance contrast) of a sample with 5-nm GaAs in the [110] direction. We can identify the Al_0.33_Ga_0.67_As barriers by their different material contrast as well as the mesoscopic structure by the increasing thickness of the GaAs layer between the AlGaAs barriers. **d** HAADF-STEM cross-section image of a sample with 5-nm GaAs in the [1–10] direction. The cut is not exactly in the middle as indicated in (**a**). We can observe the initial hole and its mound structure

This detailed study allows us to draw a complete picture of our mesoscopic GaAs structure after capping with a top 20-nm Al_0.33_Ga_0.67_As/5-nm GaAs structure. We summarize in the Fig. 3a the hole and mound evaluation in a schematic illustration. The initial GaAs structure has a well-defined, facetted, slightly off-centered hole surrounded by a GaAs mound. It does not significantly change the size after covering with AlGaAs as seen by direct comparison of the two-scale top view illustrations in Fig. 3a. Only the hole develops from a facetted structure to a more elongated structure. The top view of the MGS illustrates the position of the hole and its shape as determined from the template. Hereby, it is important to point out that the MGS is far more extended than the hole in the [1–10] direction, whereas the initial hole is larger than the mound in the [110] direction. We expect that two regions of the MGS can contribute to the optical emission: the deep hole marked mesoscopic GaAs structure—deep central hole (MGS-D) and the shallow side regime marked mesoscopic GaAs structure—shallow side structure (MGS-S). The depicted cross-section illustration in Fig. 3b is based on the AFM line scans taken in various stages of the sample growth. The MGS has a central deep hole (MGS-D) and lateral (left and right) elongated side structures with varying thickness (MGS-S) thinner than the center.

The assumptions for the schematic illustration of Fig. 3a, b are reinforced by the cross-section HAADF-STEM images depicted in Fig. 3c, d (positions and directions marked in Fig. 3a). For the [110] cross-section of Fig. 3c, the different layers can be distinguished by image contrast: from the GaAs substrate with the initial hole-mound structure, we can identify the subsequence layers, bottom 20-nm Al_0.33_Ga_0.67_As layer, 5-nm GaAs layer, the top 20-nm Al_0.33_Ga_0.67_As, and finally the GaAs cap. The grey scale image has been converted to a color scale for better identification of the different materials. A careful inspection of the hole in the GaAs substrate reveals the slight asymmetry of the hole-surrounding mound structure observed in Fig. 1a. Furthermore, it is clear from the STEM image that the Al_0.33_Ga_0.67_As layer precisely follows the morphology of the hole, simply reproducing the structure corroborating the explanation of the AFM results. As seen in the illustration and confirmed by careful examination of the STEM image, the top mound of the MGS is smaller in the [110] direction than the underlying hole structure. High-resolution TEM (not shown) demonstrats a perfect epitaxial crystal structure of the MGS.

The [1–10] cross-section is depicted in Fig. 3d. We can identify the initial hole with its surrounding mound exhibiting a total length of 425 nm. The mounds are slightly asymmetric appearing higher to the left, but extending far more to the right. In the middle, the deep hole (MGS-D) can be seen. One can recognize the two 20-nm Al_0.33_Ga_0.67_As layers that follow the underlying surface morphology and forming the extended MGS. Interestingly, we observe that the sandwiched GaAs layer becomes significantly thinner in the region over the mound reducing the thickness from 5 nm to ca. 1 nm. Due to the off-centered position of the cut, the top mound of the MGS is hardly visible, but its presence is revealed by careful inspection of the TEM image.

**Fig. 4:**
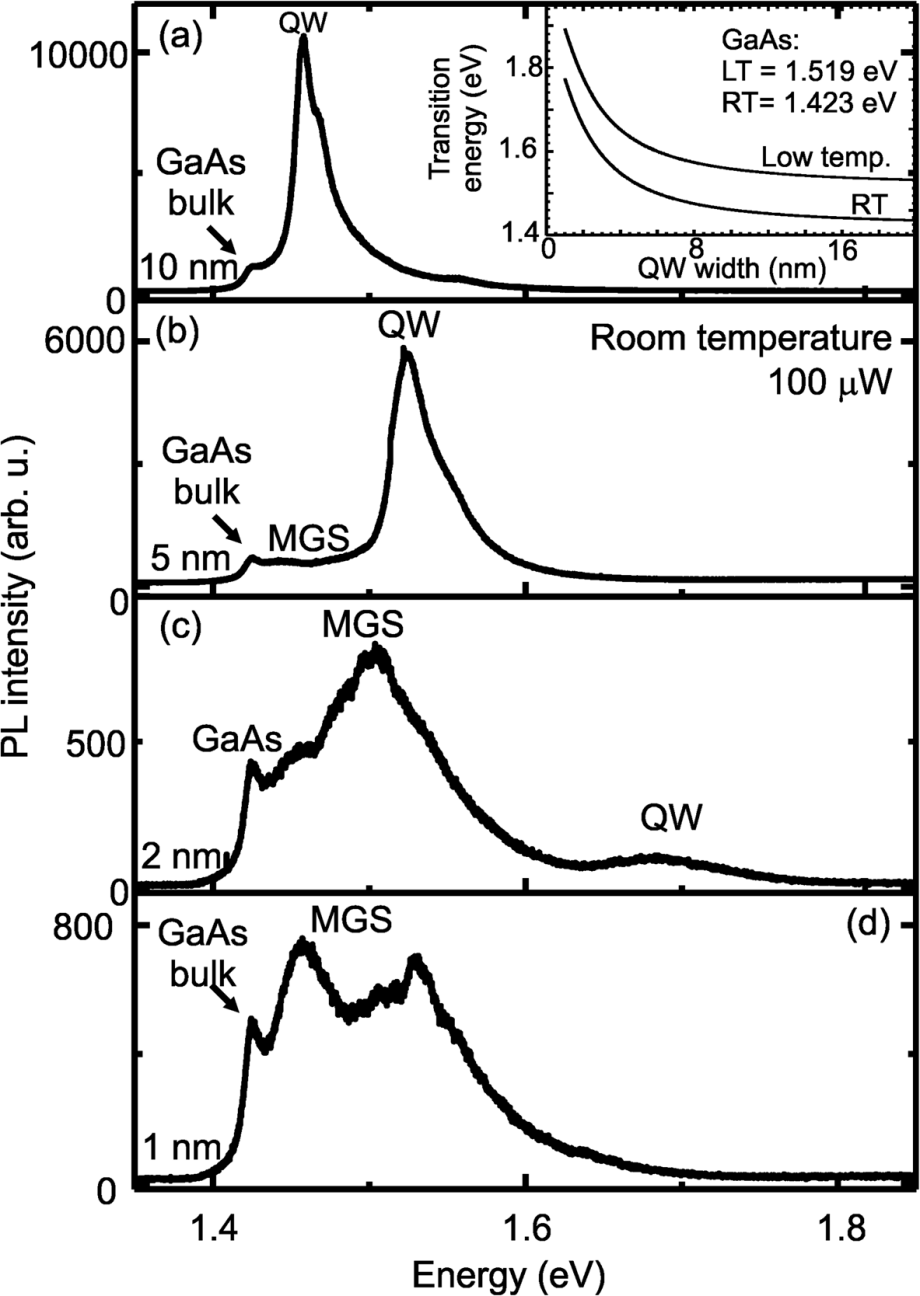
**a**–**d** Room temperature μ-PL spectra for samples with different filling of the hole-mound structures with 10-, 5-, 2-, and 1-nm GaAs layer. All samples were measured with the same integration time (30 s). The inset depicts the calculated QW transition energy versus the QW thickness

From these two HAADF-STEM cross-section images, it is clear that the GaAs filling produces the MGS with a total height of ca. 15 nm from the bottom to the top Al_0.33_Ga_0.67_As layer right over the hole-mound structure of the initial template. We ascribe this preferred GaAs nucleation inside the hole to a modulation of the chemical potential of the surface due to the curvature of the hole [41, 42]. Such curvatures and holes are effective preferred sinks for material diffusion on the top of a surface [42, 43]. The last Al_0.33_Ga_0.67_As and GaAs cap deposition follows again the mound shape of the mesoscopic structures and transfers the morphology to the surface. It is worth pointing out that away from the hole, the interface between the GaAs and the Al_0.33_Ga_0.67_As is sharp and the GaAs has reached the nominal thickness of 5 nm expected for the sample. This confirms our previous estimation that no significant amounts of deposited material are needed to fill the holes. To study the optical properties of the MGS, we carried out room and low temperature μ-PL measurements on capped samples. Figure 4a–d show the μ-PL spectra at RT using a laser power of 100 μW and a 20× objective lens, for 10-, 5-, 2-, and 1-nm GaAs deposition to fill the hole-mound structure. The spectrum depicted in Fig. 4a (10-nm GaAs deposited on top of the hole) is dominated by a peak at 1.45 eV, which is attributed to the electron-heavy hole recombination of the 10-nm-thick GaAs QW of the flat area in between the MGS. Beside this main peak, we can identify a small shoulder at 1.46 eV corresponding to the electron-light hole recombination in the QW. The small peak at 1.42 eV corresponds to the GaAs band-to-band recombination.

The inset in Fig. 4a shows the calculated emission energy of an Al_0.33_Ga_0.67_As/GaAs/Al_0.33_Ga_0.67_As heterostructure dependent on the GaAs layer thickness for low temperature (upper curve) and room temperature (RT) (lower curve), based on parabolic band approximation [8]. We find a strong confinement for small thicknesses starting from 1.65-eV emission energy at RT (1.85 eV at low temperature) for 2 nm and then reducing to 1.48-eV emission at RT (1.58 eV at low temperature) for ca. 10-nm thickness. Deep structures around 15-nm GaAs thickness should show an emission at ~1.44 eV at RT (~1.55 eV at low temperature), very close to the GaAs band gap recombination. We will use this calculation to assign the origin of observed PL peaks to different areas of the sample and inside the MGS.

Reducing the GaAs thickness, we obtain the spectrum depicted in Fig. 4b. A blue-shift of the peak energy of the 5-nm QW is observed as expected theoretically. Furthermore, an elongated shoulder between the QW and the GaAs bulk emission is now observed. We attribute this feature to the optical response of the MGS, which is expected to have a broad emission due to the height distribution of the GaAs layer. This is the first hint that the crystal quality of our mesoscopic system is good enough to allow room temperature optical experiments. Further reduction of the GaAs layer thickness confirms our assumption that the MGS exhibits an optical emission at room temperature, as shown by the spectrum in Fig. 4c, d. The PL spectra of both samples (2- and 1-nm GaAs layer) show broad emission bands between the GaAs bulk and the expected QW emissions. The inversion of the intensity ratio between the MGS and the QW indicates that carries excited into the QW diffuse rapidly into the MGS and recombine there—to a degree where the QW emission vanished and only a signal from the MGS between 1.43 and 1.6 eV is visible as observed in the spectrum shown in Fig. 4d.

**Fig. 5:**
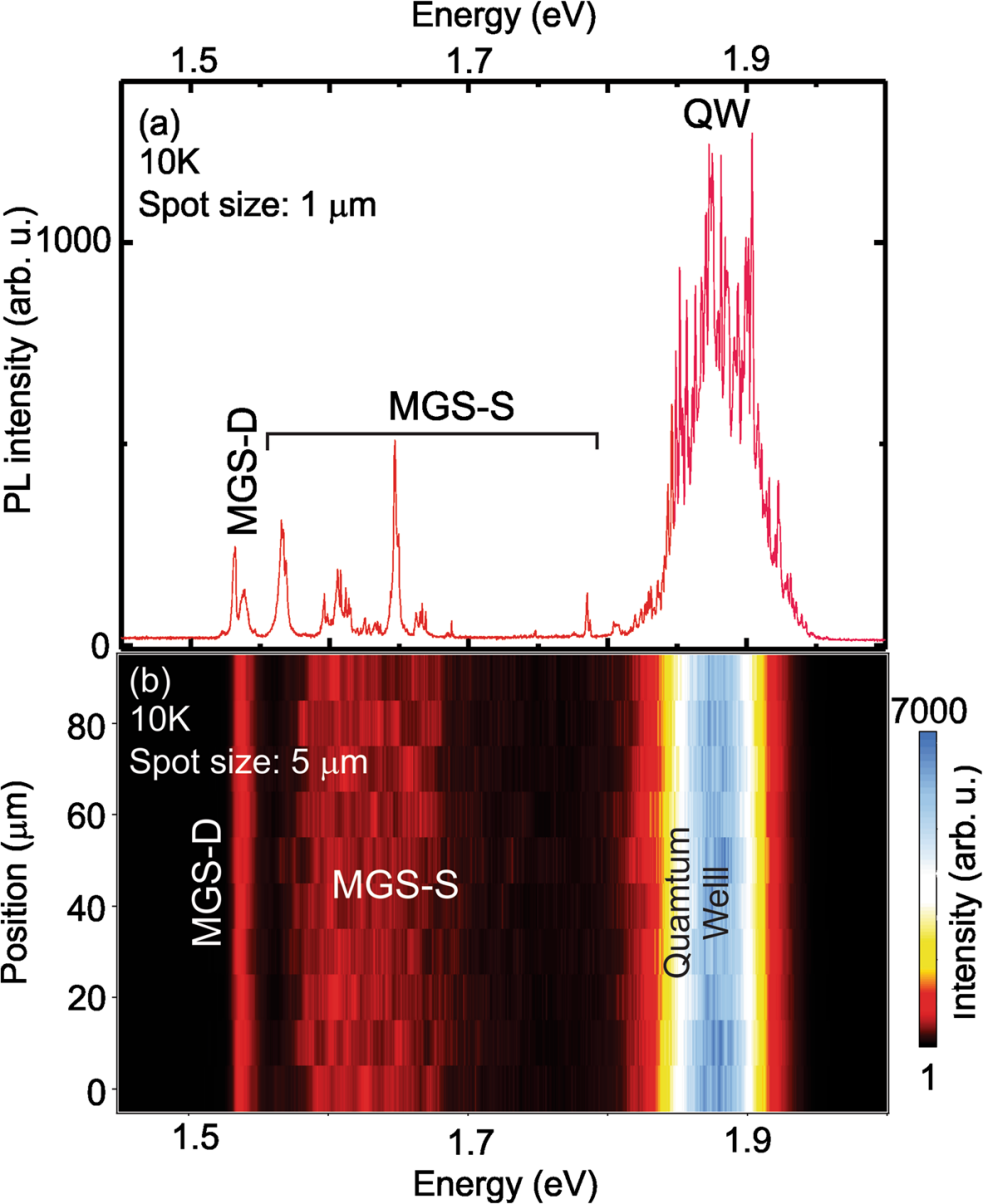
**a** μ-PL spectrum obtained with a 100× times objective (spot diameter ca. 1 μm). **b** μ-PL intensity map with a large spot size (5 μm) in steps of 20 μm along a straight line along sample with 1 nm GaAs filling of the hole-mound structures at 10 K (note the logarithmic scale of the color map, letting some of the peaks appear smaller)

To better understand the unique features of the MGS and to separate the different areas of the MGS, we carried out μ-PL at low temperature for the MGS with 1-nm GaAs active layer. Figure 5a shows a μ-PL spectrum of a single MGS obtained at 10 K using a 100× objective (spot diameter ca. 1 μm). With this spot size, only one or two MGS structures contribute to the optical emission. We observe a strong and broad peak centered at 1.88 eV and several sharp features in the region between 1.54 and 1.72 eV. In a spectral map obtained using 20× objective (Fig. 5b), it is evident that the region itself splits into two regimes.

Figure 5b displays a μ-PL spectra map obtained along a straight line in steps of 20 μm along an arbitrary sample direction. The spectra were acquired at 10 K with 500 nW laser power. In this case, several MGS contribute to each spectrum at each position. We can identify clearly three regions inside the map arising from different parts of the sample: For all the positions of the sample, a strong signal with a central peak position of 1.88 eV, as in Fig. 5a, is observed. Furthermore, a peak around 1.54 ± 0.01 eV is visible over the completely probed sample range. Finally, a broader peak constituted of several small features changing the exact peak position over the sample is seen in an energy range between 1.58 and 1.72 eV.

Using the calculated optical emission energy plotted in the inset of Fig. 4a, we can assign these peak positions to typical thickness and therefore locations of the sample. The highest energy emissions observed at 1.88 eV are attributed to the ca. 1-nm-thick GaAs QW formed by the material surrounding the MGS. This emission band is composed by sharp lines coming from the roughness of the QWs, forming localized exciton states [13, 14]. It is worth pointing out that this position is in good agreement with the expected position of the QW and indicates that our estimation of 1.5 ML GaAs to fill the holes is the upper limit, and no significant amount of deposited material migrates into the hole. The peak around 1.54 eV—splitting into a doublet for a single MGS as shown in Fig. 5a—is assigned to the 15- to 19-nm-deep hole (MGS-D). Finally, the single features between 1.58 and 1.72 eV correspond to the GaAs thickness smaller than the 15 nm of MGS-D and larger than the 1-nm QW. Therefore, we attribute them to the elongated mounds named MGS-S in Fig. 3a, b. These elongated structures have several closely spaced states from the varying thickness between 4 and 6 nm of the elongated MGS sides.

Whereas the ensemble of single peaks arising from the MGS-S region is strongly dependent on the sample location, the single peak assigned to the deep hole (MGS-D) is practically stable with only small energy shifts among them. The large GaAs thickness of the MGS-D explains the invariance of its emission energy among the single structures—for a small variation in the thickness the confinement energy variation is very small. In the case of the MGS-S, the thickness varies between 4 and 6 nm. Therefore, the transition energy shift is noticeable for any fluctuation of the thickness (see also the slope for the different thickness in the inset of Fig. 4a). It is important to point out that the optical spectrum of the MGS is different from the ones observed for GaAs quantum dots made by different methods, which exhibited normally only a single line [7, 8, 12, 23]. Indeed, we observe a combination of a single dot line (MGS-D) surrounded—and may be laterally coupled—by thickness fluctuation dots known from QWs [13–15]. Hence, the MGS as a whole forms a new kind of closely spaced thickness fluctuation quantum dot ensemble. Hereby, the single dots composing the ensemble are easily identifiable spectroscopically, but the fluctuations in the dots formed in the MGS-S are hard to identify structurally as they are not clearly visible in the TEM cross-sections of Fig. 3c, d.

**Fig. 6:**
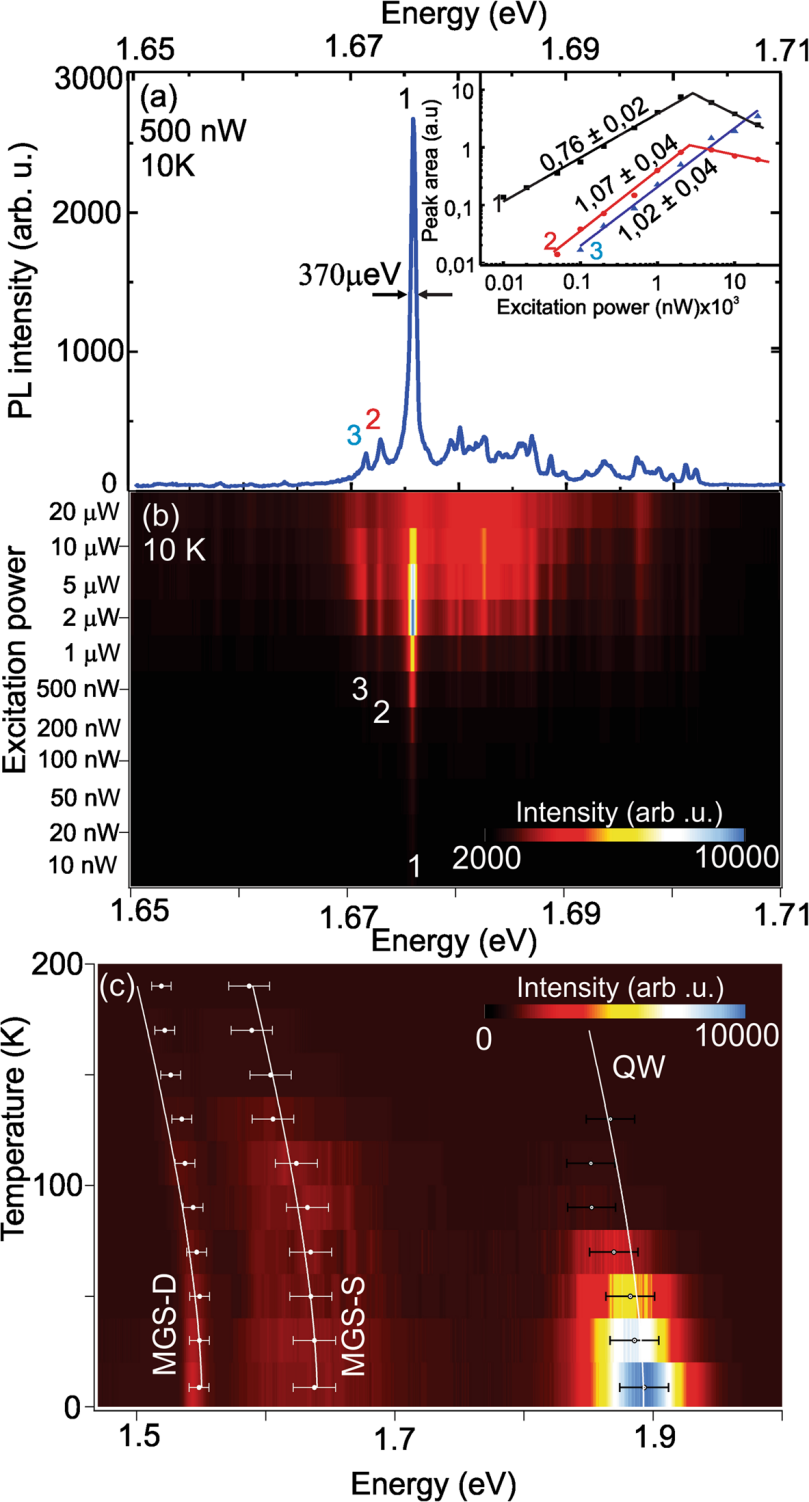
**a** μ-PL spectrum of a single structure obtained in the energy range of the MGS-S (1.65 eV and 1.71 eV) at 10 K. Three distinguished features are identified and marked with 1, 2, and 3. The linewidth of the peak 1 is ca. 370 μeV. The inset shows the integrated PL peak intensity of the marked peaks as function of laser power. **b** Power dependence map for the spectral region shown in (**a**). The analysis of the marked peaks is depicted in the inset of (**a**). **c** Temperature dependence of the PL spectra. We can follow the energy shift of three bands: QW, MGS-S, and MGS-D. Continuous line curves mark the shift of the band gap following the Varshni formula with parameters for GaAs

It is worth investigating the observed single feature ascribed to the fluctuation dots of the MGS-S in some detail. Figure 6a shows one of the single optical features seen in Fig. 5a with small spectral range corresponding to the MGS-S emissions. The spectrum is obtained using a 100× objective (ca. 1-μm spot size) with an excitation power of 500 nW at 10 K. The spectrum is dominated by a sharp peak at 1.676 eV exhibiting a full width at half maximum of 370 μeV. It is worth pointing out that we have observed features with smaller linewidth in our samples down to the resolution limit of our setup (ca. 180 μeV). Beside this central peak, two features at slightly lower energies (1.674 and 1.672 eV) are marked. Furthermore, a rather broad, underlying background between 1.669 and 1.702 eV is visible due to the relative high power used in this specific measurement.

To identify the type of recombination mechanism of three arbitrarily picked but clearly identifiable peaks marked 1, 2, and 3 in Fig. 6a, power dependent PL is carried out. The obtained PL spectra are plotted in Fig. 6b as a map with the photon energy on the abscise and the excitation power of the laser (from 10 nW to 20 μW) as ordinate. At low excitation powers of 10 nW, only the peak 1 at 1.676 eV is observed. With increasing laser power, the two other lines at 1.674 and 1.672 eV are becoming visible from ca. 100 nW on. With further increase of the excitation power, the lines are more intense until two of the peaks start to saturate—peak 1 at 5 μW and peak 2 at 10 μW. With increasing power, also the broad background feature and small lines at higher energies get more pronounced. At 20 μW, the broad background dominates the PL spectrum.

The peak integrated PL intensity *I* follows normally the formula *I = P* ^*n*^ [44], where *P* is the laser excitation power and *n* an exponent related to the recombination mechanism. Generally, *n* around 1 indicates a free and bound exciton recombination process and *n* ≥ 2 attributed to other recombination, e.g., biexcitons [15]. The inset in Fig. 6a shows the integrated PL intensity as a function of the laser excitation power for the peaks 1, 2, and 3 marked in Fig. 6b, c. Regression lines were fitted to these values to determine the slope. A saturation and a decrease in the peak area are observed for peaks 1 and 2 as seen by the abrupt change of the slope and indicated by the second fitted line. For all three peaks, we determine an exponent in the double logarithmic plot close to 1. Therefore, we conclude that all lines arise from single exciton recombination and we do not observe other process in our MGS. These excitonic lines are closely spaced in energy offering a unique system of closely spaced energy states for fundamental optical investigations [26, 27, 30, 33].

Finally, Fig. 6c depicts in a map the temperature-dependent PL intensity of 1-nm GaAs sample as a function of energy (abscissa) and temperature (ordinate) measured with 20× objective. Again, we can identify the three main contributions at low temperature (8.5 K): QW (1.88 eV), the MGS-S (1.58 to 1.72 eV) and the MGS-D (1.55 eV). Since in this measurement we have used low laser power, the map shows the spectra development only in the range from 8.5 to 190 K. With increasing temperature, the intensity of the QW decreases quickly and it shifts to lower energies. A similar trend is observed for both MGS-S and MGS-D emission. Hereby, the intensity drop is slower than for the QW—the QW is hardly visible for temperatures over 150 K, whereas we can identify the weaker optical response of the MGS-S and the MGS-D structures up to 190 K. The intensity behavior suggests the carrier transference from QW to MGSs.

To quantify these red shifts, their peak position is determined for each temperature by fitting a Gaussian to the peak and marked by white crosses in the map shown in Fig. 6c. Whereas the MGS-S and MGS-D exhibit a continuous red shift, the QW shows a non-continuous behavior by reaching a maximal red shift at 100 K followed by a red shift reduction for higher temperatures. We would expect that the red shift follows mainly the temperature dependence of the GaAs band gap *E*. Therefore, we have calculated the temperature *T*-induced shift using the Varshni formula *E = E*_*g*_ *-(AT* ^*2*^ *)/(B + T)* with the *A* = 5.4 × 10^−4^ eV/K, *B* = 199 K and *E*_*g*_ as the peak position for the lowest measured temperature using GaAs parameters [45]. The expected curves are inserted into the map in Fig. 6c for the three peaks. We find that a good fit of the MGS-S temperature behavior, whereas the QW partly deviates from the expected curve and recovers for higher temperatures. We ascribe the deviation of the QW by carrier transfer between localized states in the QW via continuum states up to 100 K, where the net emission tends to the low energy states, and above that, it is due to the thermal distribution reflecting the density of localized states. Finally, also the MGS-D departs from the expected temperature behavior. We ascribe this to a coupling of the MGS-S dots to the MGS-D ones. Similar to the QW where carries start to relax into the dots, carries from the shallow regions are able to relax into the deep hole shifting the emission energy of the MGS-D. This is a strong indication that the MGS assembles an optical system composed of single closely spaced excitonic lines, which are coupled inside the mesoscopic structures.

## Conclusions

In summary, we demonstrated that by the combination of Ga-assisted deoxidation and local hole etching at room temperature optical active mesoscopic GaAs structures (MGS) with a maximal length up to 1.2 μm × 0.2 μm can be fabricated. We investigated the overgrowth behavior of a facetted, initial hole template with AlGaAs. We engineered a hole-mound-like structure with an aspect ratio of the middle hole of 0.8 in [1–10] to [110] direction, a hole depth between 15 and 19 nm and a mound elongation of 800 nm × 200 nm in the [1–10] to [110] surface direction. In a systematic study, the hole-mounds were filled with GaAs and MGS were formed with an aspect ratio of up to 9 for a sample with 10-nm GaAs filling. These detailed morphological studies by AFM in conjunction with HAADF-STEM results allowed us to develop a detailed cross-sectional image of the MGS. The MGS were finally capped with AlGaAs/GaAs to investigate their optical emission. We found room temperature luminescence for our entire sample set. Low temperature μ-PL investigations allowed the identification of three main contributors: the QW formed by the flat area around the MGS, the elongated mounds of the MGS, and the deep hole inside the MGS. μ- PL of a single MGS revealed that the signal is composed of sharp features, which were identified as energetically closely spaced excitonic lines with the single line exhibiting a linewidth down to 180 μeV. The temperature development of the MGS spectra indicated a charge transfer from the QW regions to the elongated MGS.

Our MGSs are therefore strain-free mesoscopic optical emitters that are composed out of a single classical GaAs dot surrounded by coupled thickness fluctuation dots. Similar systems have attracted some considerable attention due to the tunability of the optical properties by electrical or mechanical means and potential use as q-bits in quantum computing [25, 32, 46]. Furthermore, it is interesting to discuss the use of the MGS as single photon sources as structures like AlGaAs/GaAs fluctuation quantum dots and filled hole structures are already being used for this propose [8, 47, 48]. One of the current challenges in single-photon emission research is to achieve efficient broadband collection. Our structures present sharp emission lines, which span approximately over 200 meV and could potentially be explored in that direction. The existence of closely spaced neighboring states is an advantage of our structure. Whereas in common experiments using multiple single photons, closely spaced states of different emitters have to be tuned with sophisticated experimental techniques [26, 29], our MGS provide them naturally. A possible influence of nearby levels on the efficiency of single photon emission in our MGS, however, must be further investigated.

## Abbreviations

AFM: Atomic force microscopy
BEP: Beam equivalent pressure
FIB: Focused ion beam
HAAD-STEM: High-angle annular dark-field scanning electron transmission microscopy
MGS: Mesoscopic GaAs structure
MGS-D: Mesoscopic GaAs structure—deep central hole
MGS-S: Mesoscopic GaAs structure—shallow side structure
QW: Quantum well
RHEED: Reflective high-energy electron diffraction
RT: Room temperature
TEM: Transmission electron microscopy
μ-PL: Microphotoluminescence
